# Clinical, echocardiographic and hemodynamic predictors of right heart failure after LVAD placement

**DOI:** 10.1007/s10554-021-02433-7

**Published:** 2021-10-18

**Authors:** M. Stricagnoli, C. Sciaccaluga, G. E. Mandoli, L. Rizzo, N. Sisti, H. S. Aboumarie, G. Benfari, L. Maritan, C. Tsioulpas, S. Bernazzali, M. Maccherini, B. M. Natali, M. Focardi, F. D’Ascenzi, M. Lisi, S. Valente, S. Mondillo, M. Cameli

**Affiliations:** 1 Section of Cardiology, Department of Medical Biotechnologies, University of Siena, Siena, Italy; 2 Adult Intensive Care Unit, Royal Brompton and Harefield NHS Foundation Trust, London, UK; 3 Department of Cardiovascular Diseases, University of Verona, Verona, Italy; 4 Department of Cardiac Surgery, University Hospital of Siena, Siena, Italy

**Keywords:** Right heart failure, LVAD, Predictors, Echocardiography, Strain

## Abstract

Right ventricular failure (RVF) after left ventricular assist device (LVAD) implant is associated with increasing morbidity and mortality. The aim of this study was to identify the best predictors of RVF post LVAD-implant among biochemical, haemodynamic and echocardiographic parameters. From 2009 to 2019, 38 patients who underwent LVAD implantation at our centre were prospectively enrolled. Preoperative clinical, laboratory, echocardiographic and haemodynamic parameters were reported. Overall, eight patients (21%) developed RVF over time, which revealed to be strongly related to overall mortality. Pulmonary artery pulsatility index (PAPi) resulted to be the most significant right heart catheterization index in discriminating RVF vs no RVF patients [(1.32 ± 0.26 vs. 3.95 ± 3.39 respectively) p = 0.0036]. Regarding transthoracic echocardiography, RVF was associated with reduced free wall right ventricular longitudinal strain (fw-RVLS) (− 7.9 ± 1.29 vs. − 16.14 ± 5.83) (p < 0.009), which was superior to other echocardiographic determinants of RVF. Among laboratory values, N-terminal pro-brain natriuretic peptide (NT-proBNP) was strongly increased in RVF patients [(10,496.13 pg/ml ± 5272.96 pg/ml vs. 2865, 5 pg/ml ± 2595.61 pg/ml) p = 0.006]. PAPi, NT-proBNP and fwRVLS were the best pre-operative predictors of RVF, a post-LVAD implant complication which was confirmed to have a great impact on survival. In particular, fwRVLS has been proven to be the strongest independent predictor.

## Introduction

Over the last decades there has been a progressive technological development of long-term durable mechanical circulatory support in response to a growing population with end-stage heart failure (ESHF), that has subsequently led to a surge in the implantation of such devices, particularly left ventricular assist devices (LVAD) [1, 2]. Heart transplantation remains, however, the gold standard among the few available therapeutic options but is strongly limited by the scarcity of donors and by the numerous contraindications. With this background, LVAD implant has become a standard of care for patients with ESHF due to left ventricular (LV) failure. Nonetheless, LVAD implant is a highly invasive procedure that requires careful and multidisciplinary evaluation to identify the patients that will benefit the most from this approach [3]. Although these patients' life expectancy has been increasing over the years, the post-operative complications still have a weight, among which post-implantation right ventricular failure (RVF) is a significant cause of increased morbidity and mortality [4, 5], associated with multiple organ failure, worse long-term survival and a lower functional status [6]. Hence, the possibility to predict the onset of RVF is extremely relevant when defining the eligibility for LVAD implantation and the best peri-operative management. Various attempts to stratify RVF risk have been proposed, including: clinical parameters such as the requirement for inotropic support, or the interagency registry for mechanically assisted circulatory support (INTERMACS) classification; invasive haemodynamic parameters obtained through right heart catheterization such as increased pulmonary vascular resistance, pulmonary capillary wedge pressure (PCWP) to central venous pressure (CVP) ratio, echocardiographic indices of RV function [7] and markers of end-organ dysfunction, such as blood-urea nitrogen levels [8]. However, a complete score including a combination of this indexes is still not available.

Our study aimed firstly to define the best pre-operative predictors, among clinical, laboratory, echocardiographic and invasive haemodynamic parameters, of post-LVAD RVF, and secondary to assess the impact of RVF on survival.

## Methods

### Study population

This was a prospective single-centre study, developed in the Department of Medical Biotechnologies, section of Cardiology, at University of Siena who involved patients implanted with a continuous flow (CF)-LVAD. From July 2009 to February 2019, a total of 39 patients were screened after being implanted with a CF-LVAD either as a bridge-to-transplant, bridge-to-candidacy or as destination therapy. Patients who were considered candidates for LVAD implantation, according to the criteria listed in the guidelines [9], after Heart Team discussion, in whom pre-operative hemodynamic, laboratoristic and echocardiographic parameters were available, were finally enrolled. Patients younger than 18 years old or patients with inadequate acoustic window were excluded. Only 1 patient was excluded due to a poor quality acoustic window, therefore the final population was represented by 38 patients. RVF was defined according to the INTERMACS criteria as elevated CVP with depressed cardiac index (< 2 L/min/m2) in the absence of elevated PCWP (< 18 mmHg), requirement for right VAD implantation, or requirement for prolonged (4 days–1 week) inhaled nitric oxide or inotropic therapy. The laboratory and echocardiographic parameters were obtained between 24 and 72 h before implantation, while an invasive hemodynamic assessment was performed within one month before the surgery. Pre-operative laboratory parameters included white blood count, haemoglobin, platelet count, creatinine, transaminase, bilirubin, urea, N-terminal pro-brain natriuretic peptide (NT-proBNP) and electrolytes. For each patient, the underlying cause of the end-stage HF, the aim of the use of LVAD, the INTERMACS class, the intra-operative complications and the outcomes in terms of mortality at 30 days, 12 months and 36 months were defined, as well as the cause of death. Adjudication of the clinical parameters were carried out by two cardiologists with expertise in advanced heart failure (HF), whereas the intra-operative complications were specified by the surgeons that performed the implant and finally both the cause of death, short- and long-term outcome were attested by a cardiologist and a cardio-thoracic surgeon both during hospital stay and at clinical follow-ups as provided by our center.

The study population was then divided into two subgroups, based on the onset of RVF. The investigation conforms with the principles outlined in the Declaration of Helsinki and was approved by our locally appointed ethics committee.

### Echocardiography

Transthoracic echocardiographic measurements were performed according to the current recommendations [10], using a high-quality ultrasound system (Vivid E7 and Vivid E9; GE, Milwaukee, Wisconsin) equipped with 2.5 MHz transducer. Standard echocardiographic parameters of the LV and the left atrium (LA) included: LV end-diastolic and end-systolic diameters from the parasternal long-axis, LV end-diastolic and end-systolic volumes, LV ejection fraction calculated by the modified Simpson's method, peak early (E) and late (A) diastolic transmitral flow velocities and E/A ratio, pulsed wave tissue doppler systolic (S′) and diastolic (E′, A′) velocities at both septal and lateral mitral annulus, E/E′ ratio, LA area and volume which were subsequently indexed by body mass index (BSA).

The echocardiographic assessment of RV size and function included both standard and advanced imaging parameters. In particular: mid-end-diastolic diameter obtained from an apical 4-chamber view; tricuspid annular plane systolic excursion (TAPSE), by M-mode technique, and pulsed wave tissue doppler systolic (S′) velocity at the lateral tricuspid annulus, as standard echocardiographic indices of longitudinal function; RV fractional area change (RVFAC) in 4-chamber view as an index of global RV systolic function. Furthermore, using speckle tracking echocardiography it was possible to measure free-wall RV longitudinal strain (fw-RVLS). A commercially available semi-automated 2-dimensional strain software (EchoPac, GE, Milwaukee, WI, USA) was used. Fw-RVLS was calculated by manually delineating the endocardial border of the free wall of RV, thus obtaining a region of interest of 3 segments (basal, middle, apical) [11]. After segmental tracking quality analysis and eventual manual adjustments of the region of interest, longitudinal strain curves were generated by the software for each ventricular segment and the global value was calculated by the software resulting in fw-RVLS. In patients where it was necessary to exclude some myocardial segments due to the inability to analyze it correctly, the longitudinal strain was calculated as the average value of the remaining segments.

### Right heart catheterization

Right heart catheterization was performed before LVAD implantation to obtain invasive hemodynamic parameters. Strict aseptic measures were applied and the procedure was performed under a local anesthetic. Each patient had ECG monitoring throughout the procedure. The transducers were zeroed both during the setup and before pressure recording, at the level of the mid-axillary line. Cardiac output and cardiac index were measured by the thermodilution method. Pulmonary artery catheters were used to measure pulmonary artery pressures (PAP), right atrial pressure (RAP) and the average PCWP. Starting from these parameters, it was then possible to calculate several indices, such as pulmonary vascular resistance (PVR), RAP/PCWP ratio, pulmonary artery pulsatility index (PAPi) and RV stroke work index (RVSWI) (calculations summarized below) [12, 13].

$$RVSWI = \left( {\frac{CI}{{HR}}} \right)/\left( {PAPm - RAP} \right)$$

The equation used for right ventricular stroke work index (RVSWI) calculation. CI = cardiac index; HR = heart rate; PAPm = mean pulmonary artery pressure; RAP = right atrial pressure

$${\text{PAPi}} = \frac{{{\text{PAPs}} - {\text{PAPd}}}}{{{\text{RAP}}}}$$

The equation used for Pulmonary Artery Pulsatility index (PAPi) calculation. PAPs = pulmonary artery systolic pressure; PAPd = pulmonary artery diastolic pressure; RAP = right atrial pressure.

### Statistical analysis

Group comparison for the continuous variables were expressed as mean ± SD or median and interquartile range, depending on the normalcy of distribution and compared by t-test, Chi-square or non-parametric tests as appropriate. Survival rates at 30 days, 6 months, and 3 years post-LVAD implant, were estimated using the Kaplan–Meier method. Survival distribution across groups were compared using the log-Rank test. The Cox-proportional hazards regression model was used to assess the effect of RVF unadjusted and adjusted for other clinically relevant variables on survival differences. The area under the curve (AUC) was calculated using a receiving operating curve (ROC), which was used to test the capability of each parameter to predict RVF after LVAD implant. Statistical analysis was performed with Statistical Package for Social Science, version 15.0 (SPSS, Chicago, Illinois). p < 0.05 was considered significant.

## Results

Table 1 summarizes the clinical features together with biochemical parameters of the study population. Around 89.5% of the total population received JARVIK 2000® LVAD and 10.5% HeartMate 3™ LVAD (Abott). Around 71% of patients received an LVAD as destination therapy. Of the 38 patients analyzed, 8 (21%) required early right ventricular support. Roughly 50% of the patients of both groups presented an ischemic etiology of the underlying LV dysfunction. Patients who developed RVF showed higher levels of creatinine and BUN, even though not statistically significant, and markedly increased levels of NT-proBNP [(10,496.13 pg/ml ± 5272.96 pg/ml vs. 2865.5 pg/ml ± 2595.61 pg/ml) p = 0.006], which was the only laboratory parameter which showed statistical significance as a predictor of RVF.

**Table 1:** Clinical and biochemical characteristics of the study population

	Total population (n = 38)	Patients with RVF (n = 8)	Patients without RVF (n = 30)	p value
*Clinical characteristics*				
Female sex, n (%)	3 (8)	1 (12.5)	2 (6.6)	
Age (y), Mean ± DS	63.08 ± 2.83	61.88 ± 5.87	63.4 ± 6.73	0.5
REDO, n (%)	11 (29)	3 (40)	8 (27)	
Body surface area, Mean ± DS	1.89 ± 0.24	1.82 ± 0.24	1.91 ± 0.20	0.27
HF etiology, n (%)				
Ischemic	21 (55)	4 (50)	17 (56.6)	
Non ischemic	17 (45)	4 (50)	13 (43.4)	
INTERMACS, n (%)				
4	10 (36)	1 (12.5)	9 (30)	
4FF	14 (37)	3 (37.5)	11 (36,6)	
3	9 (24)	3 (37.5)	6 (20)	
2TCS	3 (10)	–	3 (10)	
1TCS	2 (5)	1 (12.5)	1 (3.33)	
Indication, n (%)				
BTT	8 (21)	3 (37.5)	5 (16.66)	
DT	27 (71)	4 (50)	23(76.66)	
BTC	3 (10)	1 (12.5)	2 (6.66)	
ICU length of stay (days), Mean ± DS	14 ± 12	27 ± 3	11 ± 5	0.002
*Biochemical parameters*				
HB (g/dl), Mean ± SD	9.85 ± 2.25	11.86 ± 2.25	9.56 ± 4.05	0.15
WBC (k/mm^3^), Mean ± SD	10.59 ± 5.75	11.64 ± 3.65	10.08 ± 2.26	0.20
PLT (k/mm^3^), Mean ± SD	191.73 ± 60.10	224.4 ± 34.85	193.04 ± 59.81	0.26
CRP (mg/dl), Mean ± SD	1.74 ± 0.06	1.48 ± 1.07	1.82 ± 1.75	0.76
Creatinine (mg/dl), Mean ± SD	1.42 ± 0.12	1.48 ± 0.78	1.36 ± 0.85	0.72
GOT (UI/l), Mean ± SD	44.12 ± 7.07	47.71 ± 43.93	30.41 ± 15.07	0.08
GPT (UI/l), Mean ± SD	27.76 ± 30.40	38.14 ± 41.99	24.72 ± 15.45	0.16
Bilirubin (mg/dl), Mean ± SD	1.59 ± 0.14	2.9 ± 3.57	1.19 ± 1.68	0.06
BUN (mg/dl), Mean ± SD	63.00 ± 12.02	63.14 ± 30.09	58.66 ± 41.11	0.78
Glucose (mg/dl), Mean ± SD	103.95 ± 7.07	132.17 ± 70.7	96.59 ± 33.1	0.46
Sodium (mEq/l), Mean ± SD	136.42 ± 4.24	137.38 ± 3.46	136.07 ± 4.79	0.12
Potassium (mEq/l), Mean ± SD	4.20 ± 0.42	4.1 ± 0.6	4.30 ± 0.64	0.08
NT-proBNP (pg/ml), Mean ± SD	3748.92 ± 2427.49	10,496.13 ± 5272.96	2979.04 ± 2652.41	0.006

*BTT* bridge to transplantation, *BTC* bridge to candidacy, *BUN* blood urea nitrogen, *CRP* C-reactive protein, *DT* destination therapy, *FF* frequent flyer, *GOT* glutamic-oxaloacetic transaminase, *GPT* glutamic-pyruvic transaminase, *HB* hemoglobin, *ICU* intensive care unit, *INTERMACS* interagency registry for mechanically assisted circulatory support, *NT-proBNP* N-terminal fragment of pro-B-type natriuretic peptide, *PLT* platelets, *REDO* reoperative heart surgery, *RVF* right ventricular failure, *TCS* temporary circulatory support, *WBC* white blood cells

**Fig. 1:**
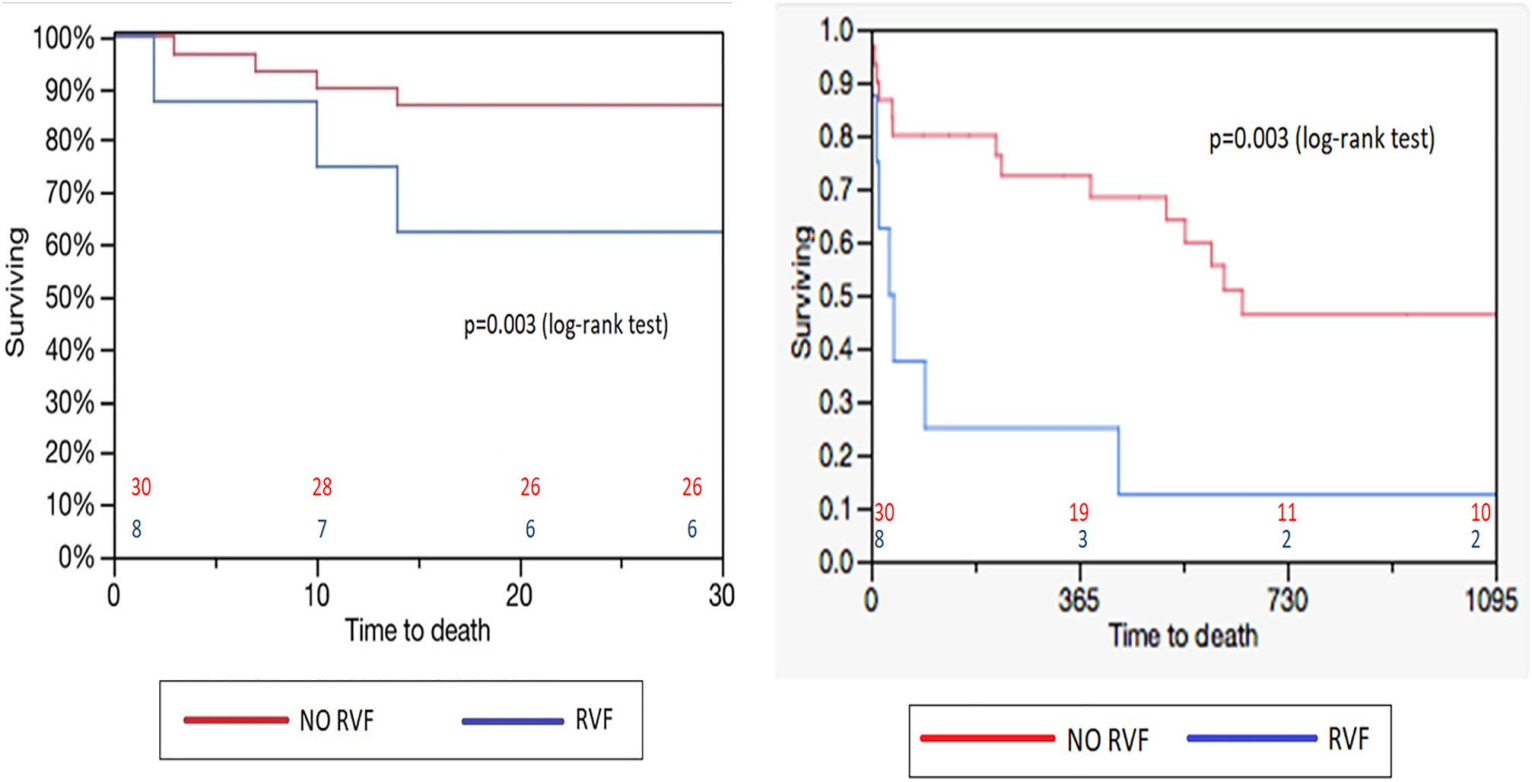
Kaplan–Meier curves. Right ventricular failure (RVF) is strongly associated with patients’ survival after LVAD implant. The figure on the left shows the difference of 30-day survival rate between patients with and without RVF, while the picture on the right shows 3-year survival rate

RVF was strongly associated with survival as shown by the Kaplan–Meier curves (p = 0.003) (Fig. 1) and the Cox proportional risk model [HR 3.42 (95% CI 1.41–8.16) p = 0.01]. RVF patients showed a 30-day survival of 25% compared to 77% in non-RVF patients, and only 1 out of 8 patients in the RVF group survived up to 3 years.

Regarding the hemodynamic parameters (Table 2), RAP was fairly high in patients who had RVF [(17.23 ± 4.06 mmHg vs. 8.07 ± 4.24 mmHg) p = 0.007] compared to the other patients, contrary to the RAP / PCWP ratio [(3.05 ± 1.70 vs. 3.31 ± 1.20) p = 0.01] and to PAPi which were lower in patients suffering from RVF [(1.52 ± 0.26 vs. 3.95 ± 3.39) p = 0.0036]. Table 3 shows correlation analysis for the most relevant hemodynamic parameters.

**Table 2:** Hemodynamic and echocardiographic parameters

	Total population (n = 38)	Patients with RVF (n = 8)	Patients without RVF (n = 30)	p value
Hemodynamic parameters				
Cardiac Index (L/min/m^2^), Mean ± SD	2.03 ± 0.21	1.66 ± 0.46	2.14 ± 0.69	0.07
PAPs (mmHg), Mean ± SD	44.83 ± 9.19	49.38 ± 12.37	43.59 ± 15.81	0.41
PAPd (mmHg), Mean ± SD	21.00 ± 6.09	23.00 ± 6.02	20.45 ± 7.08	0.35
PAPm (mmHg), Mean ± SD	29.83 ± 2.82	33.13 ± 8.81	28.93 ± 9.68	0.27
PCWP (mmHg), Mean ± SD	20.30 ± 14.84	21.88 ± 7.64	19.86 ± 7.62	0.51
RAP (mmHg), Mean ± SD	10.05 ± 2.12	17.23 ± 4.06	8.07 ± 4.24	0.007
RAP/PCWP, Mean ± SD	0.53 ± 0.30	0.82 ± 0.15	0.45 ± 0.30	0.01
PVR (term), Mean ± SD	3.22 ± 0.17	3.05 ± 1.70	3.31 ± 1.20	0.68
PAPi, Mean ± SD	3.40 ± 0.47	1.52 ± 0.26	3.95 ± 3.39	0.003
RVSWi (mmHg × L/m2), Mean ± SD	0.58 ± 0.07	0.33 ± 0.11	0.66 ± 0.48	0.06
Echocardiographic parameters				
LV-EDD (mm), Mean ± SD	69.81 ± 4.24	68.50 ± 8.59	71.07 ± 10.52	0.15
LV-EDS (mm), Mean ± SD	58.50 ± 9.19	54.75 ± 9.68	59.59 ± 12.30	0.31
RV-EDD (mm), Mean ± SD	35.56 ± 0.70	38.75 ± 7.32	34.69 ± 4.54	0.06
LVEF (%), Mean ± SD	23.84 ± 7.07	23.25 ± 5.36	24 ± 4.26	0.67
RV/LV diameter ratio, Mean ± SD	0.49 ± 0.06	0.49 ± 0.08	0.49 ± 0.01	0.6
PAPs (mmHg), Mean ± SD	48.17 ± 3.53	52.5 ± 12.94	47.08 ± 14.67	0.41
Sphericity Index, Mean ± SD	0.43 ± 0.01	0.48 ± 0.08	0.44 ± 0.87	0.21
TAPSE (mm), Mean ± SD	15.51 ± 3.53	11.88 ± 2.90	16.52 ± 4.40	0.02
RVFAC (%), Mean ± SD	39.29 ± 10.60	34.63 ± 9.98	40.59 ± 5.15	0.04
Fw-RVLS (%), Mean ± SD	− 14.31 ± 2.90	− 7.9 ± 1.29	− 15.99 ± 5.63	0.009
S′ tric (m/s), Mean ± SD	0.09 ± 0.07	0.07 ± 0.03	0.11 ± 0.03	0.05
LA area (cm^2^), Mean ± SD	31.10 ± 4.90	32.1 ± 4.81	30.76 ± 5.43	0.61
LA indexed volume (ml/m^2^), Mean ± SD	61.25 ± 8.15	69.27 ± 0.25	59.12 ± 24.27	0.31
E/E′ ratio, Mean ± SD	15.42 ± 4.46	15.50 ± 1.46	15.41 ± 8.83	0.98

*E* early transmitral velocity, *E*′ early diastolic mitral myocardial velocity, *Fw-RVLS* free wall right ventricular longitudinal strain, *LA* left atrial, *LV* left ventricular, *LV-EDD* left ventricular end diastolic diameter, *LVEF* left ventricular ejection fraction, *LV-ESD* left ventricular end systolic diameter, *PCWP* pulmonary capillary wedge pressure, *PAPd* pulmonary artery diastolic pressure, *PAPi* pulmonary artery pulsatility index, *PAPs* pulmonary artery systolic pressure, *PVC* pulmonary vascular resistance, *RAP* right atrial pressure, *RV-EDD* right ventricular end diastolic diameter, *RVF* right ventricular failure, *RVSWI* right ventricular stroke work index, *S*′ tric tricuspidal systolic myocardial velocity, *TAPSE* tricuspid annular plane systolic excursion

**Table 3:** Correlation analysis for haemodynamic parameters

Variable	B	HR	95% CI	p value
PAPs (mmHg)	0.025	1.025	0.974–1.079	0.341
PAPm (mmHg)	0.045	1.046	0.965–1.135	0.275
PCWP (mmHg)	0.035	1.036	0.934–1.149	0.502
RAP (mmHg)	0.519	1.680	1.150–2.453	0.007
PVR (term)	− 0.178	0.837	0.364–1.923	0.675
CI (L/min/m^2^)	− 1.684	0.186	0.027–1.288	0.088
RA/PCWP	4.106	60.701	2.617–1407.719	0.010
PAPI	− 1.847	0.158	0.025–0.978	0.047
RVSWI (mmHg x L/m^2^)	− 5.731	0.003	0.000–0.727	0.038

*CI* cardiac index, *PAPm* pulmonary artery mean pressure, *PAPi* pulmonary artery pulsatility index, *PAPs* pulmonary artery systolic pressure, *PCWP* pulmonary capillary wedge pressure, *PVR* (Term) pulmonary vascular resistance by termodiluition method, *RAP* right atrial pressure, *RVSWI* right ventricular stroke work index

**Fig. 2:**
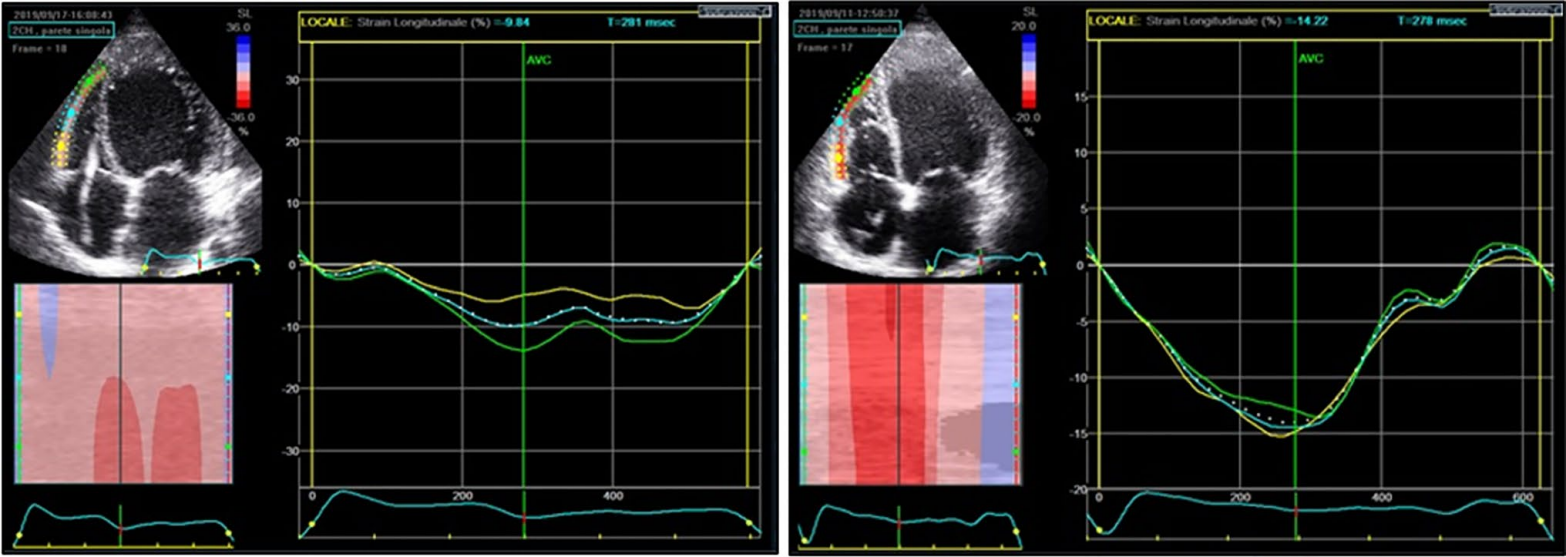
Difference in free-wall right ventricular longitudinal strain between patients with and without right ventricular failure. This picture shows the difference in free-wall right ventricular longitudinal strain (fw-RVLS) between a patient that developed right ventricular failure post-LVAD implant (on the left) and another patient that did develop this complication (on the right). In fact, it is possible to appreciate the significant difference between the two value: a higher absolute value in fw-RVLS in patients without right ventricular failure

In RVF group we found pre-operative lower TAPSE (RVF 11.88 ± 2.90 mm vs. no-RVF 16.52 ± 4.40 mm p = 0.02 OR 0.71), lower RVFAC (RVF 34.63 ± 9.98% vs. no-RVF 40.59 ± 5.15% p = 0.04 OR 0.87), reduced fw-RVLS (− 7.9 ± 1.29% vs. − 15.99 ± 5.15 p < 0.009) (Fig. 2; Table 2).

**Fig. 3:**
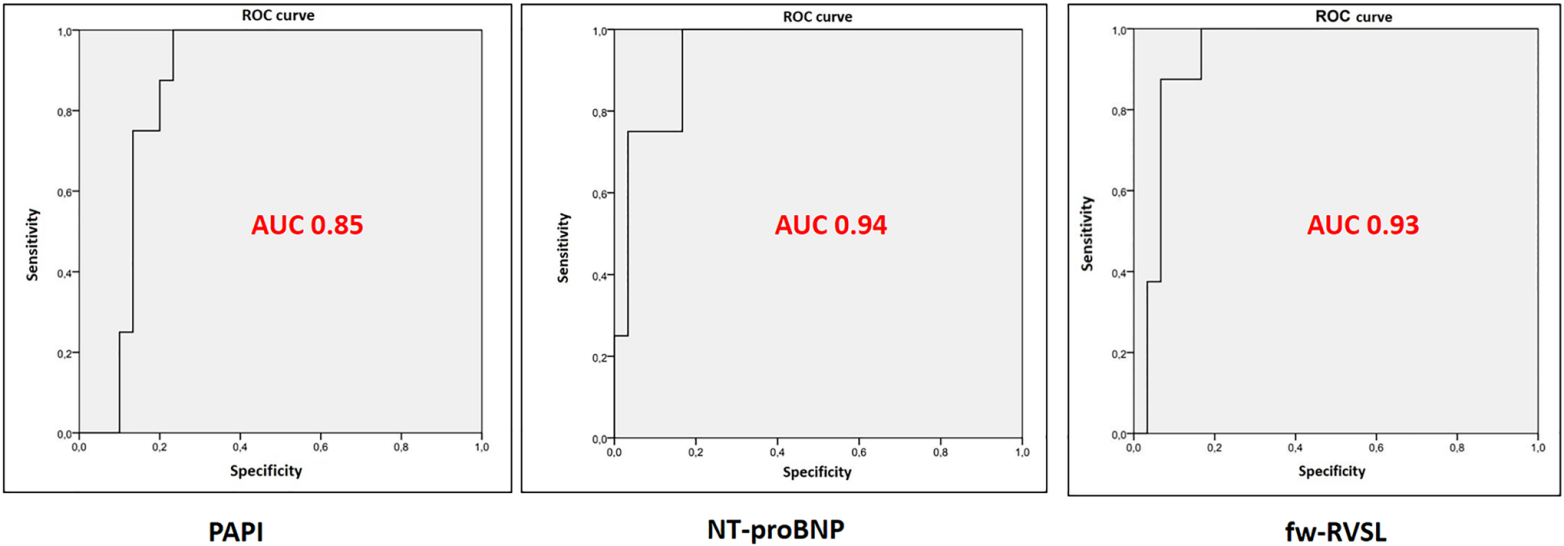
Receiving operator curves. This figure shows receiving operator curves (ROCs) of the three main parameters that was found significantly associated with the development of right ventricular failure post-LVAD implant, respectively PAPI (pulsatility arterial pulmonary index, on the left), NT-proBNP (N-terminal pro-brain natriuretic peptide, in the middle) and fw-RVLS (free-wall right ventricular longitudinal strain, on the right)

Applying ROC curve analysis to the selected variables, the predictive value was tested for all the indexes. Only 3 of them revealed an AUC > 0.80: PAPi (0.85), NT-proBNP (0.94) and fw-RVLS (0.93) (Fig. 3).

Fw-RVLS was superior to the other echocardiographic parameters and remained independently associated with RVF in a bivariate analysis that included laboratory values (p = 0.04 after adjustment with NT-proBNP) or invasive parameters (p = 0.04 after adjustment with PAPi), even when including both in the model, a trend of significance for fw-RVLS was found (p = 0.0065).

## Discussion

**Fig. 4:**
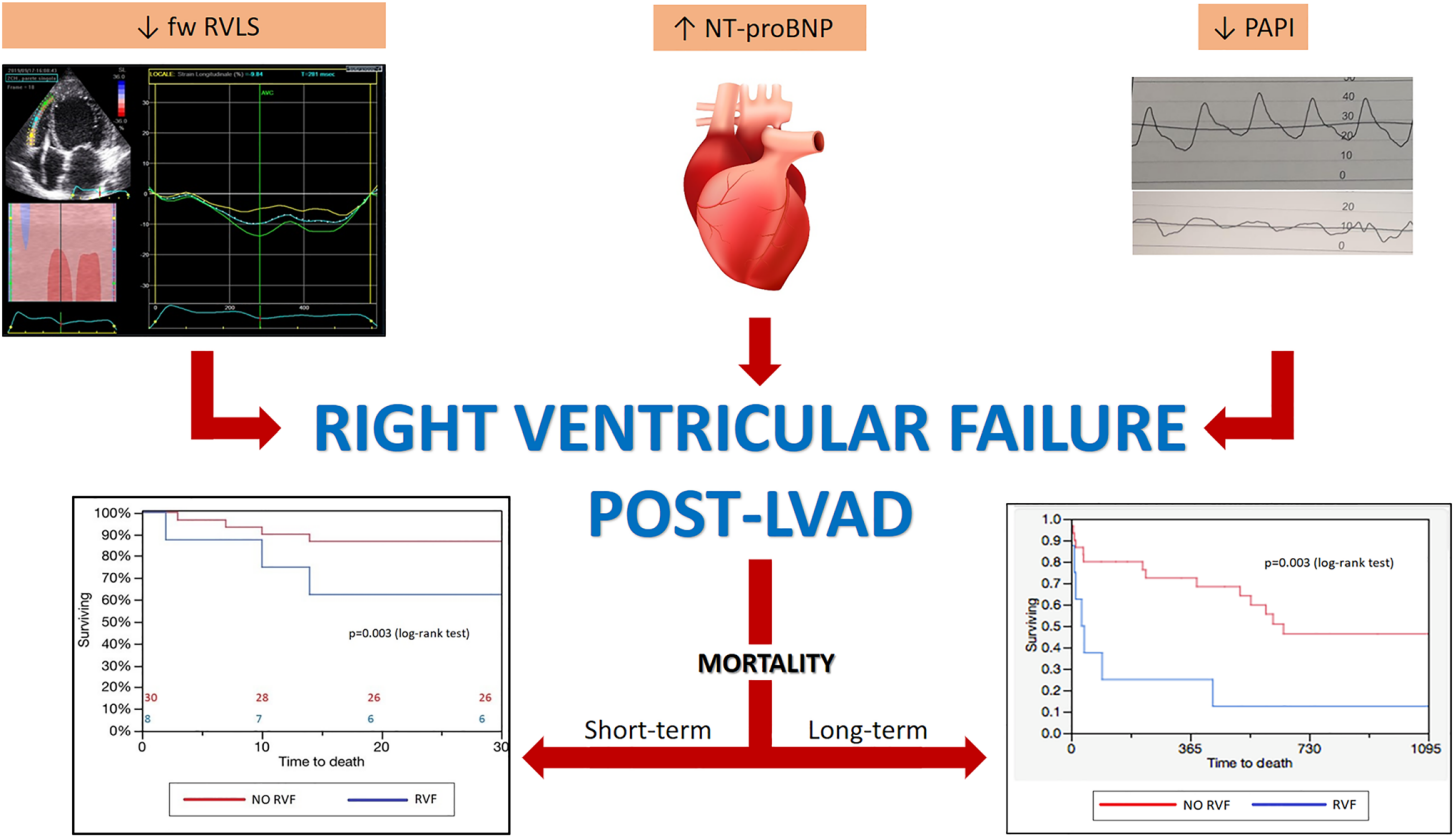
Central illustration: risk factors of right ventricular failure post-LVAD implant. This figures summarizes the findings of our study. Free wall-right ventricular longitudinal strain (fw-RVLS), pulmonary artery pulsatility index (PAPi) and N-terminal pro-brain natriuretic peptide (NT-proBNP) resulted to be the best predictors of right ventricular failure post-LVAD implant. Furthermore, the development of right ventricular failure was responsible for a significant short- and long-term mortality rate, with a great impact on post-LVAD survival

The main findings of the study can be summarised as follow: (1) RVF was strongly associated with survival; (2) RAP was fairly high in patients who had RVF, contrary to the RAP/PCWP ratio and to PAPi which were significantly lower, and among these indexes, PAPi resulted to be the most statistically significant haemodynamic parameter correlated to RVF; (3) The only laboratory parameter which showed statistical significance as a predictor of RVF was NT-proBNP; (4) Overall, PAPi, NT-proBNP and fw-RVLS, were the best pre-operative predictors of RVF post-implantation; (5) fw-RVLS resulted to be the strongest independent risk predictor for developing RVF post-LVAD implant (Fig. 4).

With a growing LVAD-eligible population, post-implantation RVF is becoming more frequently encountered in clinical practice and it represents one of the major causes of morbidity and mortality in the early postoperative period [14, 15]. Therefore, the development of strategies enabling the prediction and the prevention of this challenging complication has become increasingly relevant, and despite the numerous clinical studies and algorithms which developed over the years, post-LVAD RVF remains a dilemma. The difficulty relies on both the methodological principles and on the complexity of the pathophysiological definition of post-LVAD RVF. There is a strong rationale supporting the predictive value of a pre-existing, subclinical or manifest, RV dysfunction. As a matter of fact, in our study, all patients who developed post-LVAD RVF had some degrees of pre-operative RV dysfunction. Analyzing the pre-existing predictive scores, a high heterogeneity emerges both in the definition of RVF and in the indications for LVAD implantation. Furthermore, most of the scores were derived from old generation pulsatile flow devices and are based on monocentric studies [15–17]. A recent retrospective study by Pettinari et al. [18] applied three different risk scores to a population of 59 patients who underwent LVAD implantation and none of the scores proved to be predictive of RVF. The same was done in a study by Kalogeropoulos et al. [19] which utilized six recent predictive scores which confirms a loss of predictive power in populations different from their respective derivation cohorts.

It should also be noted that the current INTERMACS definition of RVF does not include any direct quantitative parameter of RV function. Regarding the echocardiographic evaluation of RV function, a qualitative estimation is considered a simple and robust index by some experts, even though this approach is considered of poor reproducibility, reduced accuracy and sensitivity in detecting clinically significant changes. Hence the need to identify quantitative and reproducible parameters to be included in a pre-implantation evaluation process.

The primary objective of our study was to identify parameters which were capable of predicting the onset of post-LVAD implantation RVF by analyzing echocardiographic, laboratory and hemodynamic data. Patients were divided into two subgroups based on the development of RVF. The two subpopulations were similar in age, BSA and HF aetiology. The INTERMACS profiles did not show substantial differences between the two groups, although patients who later developed RVF tended to have lower INTERMACS class.

The mean length of stay in the intensive care unit was significantly longer in the RVF group. Analysing the laboratory data, NT-proBNP proved to be the only parameter with significant predictive power (p = 0.006). In literature, results regarding the role of NT-proBNP value in this setting are less consistent. In fact, besides the well-known prognostic value of this biomarker in HF patients, a recent meta-analysis showed that patients that presented RVF post-implant had the tendency to have higher levels of NT-proBNP, even though high heterogeneity was found among the studies [20]. Our study confirmed the relatively great variability of NT-proBNP values in this subset of patients, even though its predictability of RVF was fairly high. Surprisingly enough, our results did not show a significant difference between the two subgroups with regards to renal function, which is a well-known prognosticator, together with bilirubin and haemoglobin [14, 15, 17], which also did not reach statistical significance in our population.

Cardiac catheterization measurements are particularly useful to define RVF [21]. On one hand, patients who developed RVF showed lower values of RVSWI, although this parameter did not reach the expected statistical significance, probably due to the small sample of the study. On the other hand, RAP, RAP/PCWP ratio and PAPi were statistically significant. In particular, PAPi showed the greatest predictive power. This index was initially developed as a predictor of RVF in the setting of acute inferior myocardial infarction to identify patients requiring RV mechanical support. Kang et al. [22] applied it to a population of 83 patients who were candidates for a continuous flow LVAD implant and in this setting PAPi emerged as a strong predictor of post-operative RVF. The difference between the PAPI and the RAP/PCWP ratio is that the first is less subject to the influence of the left heart, thus representing a 'pure' right-sided heart measurement that gauges RV systolic effectiveness, which might partially explain the relatively high AUC of this parameter with regards to RVF prediction.

There is an increasing interest in the preoperative morpho-functional echocardiographic evaluation of the RV, due to the increasing attention to its prognostic role in HF. In fact, its relevance has been widely recognised not only in patients with reduced LV ejection fraction but also in HF with preserve ejection fraction, highlighting how its role goes beyond ejection fraction. For instance, a recent study showed that in patients with acute HF, RV systolic impairment together with RV-pulmonary artery uncoupling is associated with lung congestion both on admission and on discharge, the latter being a strong prognostic factor [23]. A high number of echocardiographic parameters has been associated with RVF [24–27]. The most significant in our study was the fw-RVLS (p = 0.009), superior to other echocardiographic RVF determinants. Furthermore, its independent predictive power was sustained even in a bivariate model including clinical (p = 0.009 after adjusting for NT-proBNP) or invasive parameters (p = 0.003 after adjusting for PAPi). Even after forcing both NT-proBNP and PAPi in the model, fw-RVLS presented a trend towards RVF prediction (p = 0.065).

Speckle tracking echocardiography allows an objective and quantitative evaluation of global and regional myocardial function, independent of the insonation angle and the cardiac translational movements [28], through the measurement of longitudinal strain. There is growing evidence supporting the role of strain imaging for RVF prediction [29]. Indeed, in a retrospective study based on 117 patients undergoing LVAD implantation [30], fw-RVLS predicted RVF with 76% specificity and 68% sensitivity using a cut-off value of − 9.6%. In this study, the reported incidence of RVF was about 40%. In another cohort of 68 patients undergoing elective LVAD implantation [31], fw-RVLS resulted to be significantly compromised pre-operatively (− 12.6 ± 3.3% vs. − 16.2 ± 4.3%; p < 0.001) in 24 patients (35.3%) who presented with early RVF. Furthermore, fw-RVLS has been shown to correlate with invasively measured RVSWI [32]. These evidence might support the fact that fw-RVLS showed higher AUC compared to previous work. A remarkable meta-analysis including a total population of 4428 patients with advanced HF referred for LVAD implant, found that having either moderate to severe RV dysfunction, assessed by qualitative analysis of echocardiographic images, was associated with right ventricular failure, with AUC being 0.68 [20]. Of note, only three study out of the thirty-six study included in the meta-analysis measured longitudinal strain of right ventricular free wall [32, 33]. As mentioned above, this parameter has been gaining strong relevance in heart failure patients, showing a strong prognostic role in patients referred for LVAD implant. Besides the relatively small sample population of our investigation, free wall right ventricular longitudinal strain was performed by a cardiologist with high expertise in speckle tracking echocardiographic, which might have positively affected the results due to a higher accuracy in the measurement.

Parameters such as RVFAC, TAPSE and Tissue Doppler-derived systolic velocity (S′) of the tricuspid valve annulus have shown lower predictive power. This could be related to the fact that both TAPSE and the systolic velocity (S′) of the tricuspid annulus are regional parameters, both dependent on loading conditions and insonation angle [34]. Moreover, the translational motion of the heart and the tethering by the adjacent impaired myocardial segments can produce velocities that are not representative of the performance of the interrogated segment. RVFAC represents a two-dimensional approach to the complex geometry of the right ventricle, it is a completely manual method which does not provide information on wall deformation. Finally, technical issues could make this index less reproducible than fw-RVLS, such as heavy RV trabeculations and pacemaker or defibrillator-related artefacts in patients with advanced HF.

Finally, our study confirms the strong impact of RVF on early mortality: all patients in which RVF occurred developed this complication in the immediate postoperative period. Comparing the Kaplan–Meier curves of the two groups, a significant survival difference emerged, already after the first month of LVAD placement.

Our study has some limitations which include its single-centre design and relatively small sample size, which precluded us from identifying cut-off values for each significant predictive variable. Finally, the haemodynamic evaluation was performed near the time of LVAD implantation for most of the patients, but not in the whole population, which might have affected its predictive power.

## Conclusions

The results of our study confirm that RVF has a great impact on survival post-LVAD implantation, which highlights the necessity for having reliable predictors to best avoid this complication. Among the analysed parameters, PAPi, NT-proBNP and fw-RVLS, were the best pre-operative predictors of RVF post-implantation. Particularly, fw-RVLS has been proven to be the strongest independent risk predictor for developing RVF. Hence, these three indices might significantly improve the definition of eligibility for LVAD implantation as well as the clinical peri-operative management.
